# Parallel Mixed Image Encryption and Extraction Algorithm Based on Compressed Sensing

**DOI:** 10.3390/e23030278

**Published:** 2021-02-25

**Authors:** Jiayin Yu, Chao Li, Xiaomeng Song, Shiyu Guo, Erfu Wang

**Affiliations:** Electrical Engineering College, Heilongjiang University, Harbin 150080, China; yujiayin0920@163.com (J.Y.); lichao_6633@163.com (C.L.); 18846433840@163.com (X.S.); 2181238@s.hlju.edu.cn (S.G.)

**Keywords:** mixed image, compressed sensing, parallel transmission, chaotic matrix

## Abstract

In the actual image processing process, we often encounter mixed images that contain multiple valid messages. Such images not only need to be transmitted safely, but also need to be able to achieve effective separation at the receiving end. This paper designs a secure and efficient encryption and separation algorithm based on this kind of mixed image. Since chaotic system has the characteristics of initial sensitivity and pseudo-randomness, a chaos matrix is introduced into the compressed sensing framework. By using sequence signal to adjust the chaotic system, the key space can be greatly expanded. In the algorithm, we take the way of parallel transmission to block the data. This method can realize the efficient calculation of complex tasks in the image encryption system and improve the data processing speed. In the decryption part, the algorithm in this paper can not only realize the restoration of images, but also complete the effective separation of images through the improved restoration algorithm.

## 1. Introduction

Image information is an important information carrier. It has become an indispensable part of People’s Daily production and life [1]. With the increasing volume of data, the security and efficiency requirements of image information transmission are also increasing [2]. The research on image encryption algorithms is deepening. Effective encryption algorithms can resist illegal tampering, destruction, and attack [3]. Compressed sensing, as a computing method that can greatly reduce the sampling rate, has attracted extensive attention [4]. This algorithm can break Nyquist’s sampling theorem [5], improve computational efficiency, and save computational resources. Terence Tao of the University of California, Los Angeles, Emmanuel Candes of the California Institute of Technology, David Donoho of Stanford University, and Richard Baraniuk of Rice University were among the first to propose compressed sensing [6]. Due to its excellent properties, compressed sensing theory is widely used in image processing [7], Wireless communication [8], Medical imaging [9], Radar, and other fields [10]. The theory has also been awarded one of the top 10 technological advances of the year in the United States. Chen et al. designed an optical image conversion and encryption algorithm using the phase detection algorithm and the incoherent superposition algorithm, which could convert the color image to the gray image and encrypt it [11]. In reference [12], an encryption algorithm combining orthogonal coding and double random phase coding is proposed, which can compress all images into random signals and diffuse them into stationary white noise. Xiao et al. first carried out sparse processing on the image, and then carried out Arnold transform on it. When the image is measured, the watermark is embedded into the measurement quantity to get the measured value of the image with the watermark as the password [13]. At the same time, the research on compressed sensing is also underway in China. Researchers from The Chinese Academy of Sciences designed an experimental device for superresolution imaging based on compressed sensing [14]. According to the theory, Chongqing University also designs a frequency-domain all-pass random filter [15]. A scholar from Beijing Jiaotong University proposed an indoor location algorithm based on compressed sensing [16]. Yang et al. proposed an image encryption algorithm based on fractional-order hyperchaotic composite system and Galois field (GF) [17]. Column cyclic shift is used in the encryption process, and GF algorithm is used to diffuse pixel values. This algorithm has good security performance. In paper [18]. A block cipher structure based on diffusion, scrambling, and S-box encryption is proposed to encrypt the quantized data, which shows excellent performance in compression performance and encryption speed.

An effective image compression encryption algorithm based on chaotic system and compressed perception is proposed in [19]. In this algorithm, measurement matrix is constructed by chaotic system to realize effective encryption of image information. Hu et al. proposed a parallel computing method, which can effectively improve the computational efficiency [20]. They used the concept of parallel transmission in his algorithm. By means of the cooperation between the construction process of nonlinear chaotic sensing matrix and the anti-pattern operation, the select-plaintext attack (CPA) can be resisted.

In the previous study, we designed a compressed sensing encryption algorithm based on a sequence signal generator [21]. It greatly expands the key space of the algorithm. The algorithm realizes secure encryption and effective decryption. At the same time, we also studied the image with noise [22]. On the basis of the above work, an image encryption algorithm that can resist noise interference is designed.

Most image information encryption is based on a single, unmixed image. However, in the actual image processing, there are many mixed image problems. For example, it is common to see a linear superposition of two images in many drawing software. These include the background and text of two images of the mixture, may also be a mixture of multiple images; Or combine several single channels into a multi-channel array to create a multi-channel array composed of multiple single channel arrays. It is also possible that image contamination due to camera shake, lens change, etc., can also lead to image blending. Some scholars have carried out research on the encryption of mixed images. In the literature [23], several small images are combined into one large image, and these image elements are disturbed by the chaotic sequence generated by the PWLCM system to obtain the ciphertext image. After decryption, the small image is segmented to get the original image. Zarebnia et al. proposed a fast and efficient multi-image encryption algorithm based on chaotic systems [24]. A chaotic sequence generated by a chaotic system is used to replace the subblocks of the combined image. In order to obtain the encrypted image, Arnold Cat Map and several operations. Including XOR and cyclic shift, are used. In the literature [25], multiple images are superposed to form 3D images. This algorithm uses chaos theory and the concept of Elgamal cryptosystem of elliptic curve to generate cryptographic images and share keys. Perform displacement and replacement operations on the 3D image to generate password data.

The difference between this paper and the above studies is that the focus of our study is on the mixed images. After the images are mixed, they cannot be separated by partitioning, but the pixel values of two or more images are mixed together. We encrypt and reconstruct this image. This article is mainly for processing mixed image to design a kind of encryption algorithm. This paper mainly designs an encryption and decryption algorithm for processing mixed images. The algorithm is based on compressed sensing framework and chaotic encryption theory. From the perspective of information security, it can improve the security of encryption on the one hand, and reduce the time of encryption and decryption to the greatest extent on the other hand. By reducing the amount of key transmission, the cost of information storage is reduced. At the same time, this algorithm makes use of the idea of parallel operation. It can greatly reduce the complexity of calculation and improve the overall efficiency of the algorithm.

In the first part of this paper, we introduce the research background of the algorithm and the research status at home and abroad. The second part describes the mathematical model of chaotic system and the mathematical representation of compressed sensing theory used in this paper. The third part describes the encryption and decryption algorithm and verifies that the algorithm can effectively recover the original signal in the state of unmixed image. In the fourth part, the mixed image is simulated and verified, and several parameters are used to analyze the effectiveness of the separated image and the security of the algorithm [26].

## 2. Chaotic System Theory and Compressive Sensing Principle

The chaotic system adopted in this paper is a Three-Dimensional chaotic system with a single fixed point proposed by [27]. Since this kind of chaotic system has a fixed point, we can analyze the stability of the system. The chaotic system with stability characteristics can enhance the stability of the whole algorithm, while the three-dimensional system has more complex dynamic behavior and can achieve higher safety performance. This design is based on the compressed sensing theory. Compressed sensing theory contains three important components, which are, respectively, the sparsity of signals, the search for appropriate transformation domain, the construction of measurement matrix and the design of reconstruction algorithm. In this paper, the original signal is represented sparsely by wavelet change to realize the signal sparsity process. The measurement matrix is generated by chaotic systems, and the sparse signal is compressed and sampled. In the reconstruction algorithm part, an OMP algorithm is adopted and improved in order to separate the mixed signals after reconstruction.

### 2.1. Mathematical Representation of Chaotic Systems

Chaotic attractors with hidden dynamical properties have attracted the special attention of mathematicians and engineers [28]. The existence of unknown hidden attractors may cause a project or system to fail. Due to the difficulty in predicting hidden attractors, there is no effective method to predict the existence of hidden attractors in the system. Ref. [29] provides a basis for further understanding the complex mechanism of chaos dynamics hidden in discrete maps. The hidden chaotic attractors in the map are explored using a computer exhaustive search [30]. A novel chaotic circuit with parallel memristor and linear inductor is studied in the literature [31]. A total of 19 different types of chaotic attractors are found by establishing mathematical models. The equilibrium point and stability of chaotic systems are analyzed by using the traditional dynamic analysis method. In addition, some special phenomena are found, such as state transitions, chaotic degradation, and multiple coexisting attractors. In this algorithm, a THREE-DIMENSIONAL chaotic mapping system is used, and the mathematical expression is shown as 2-1:

(1) $$C=\{\begin{array}{c} x\left(i+1\right)=y\left(i\right)\\ y\left(i+1\right)=z\left(i\right)\\ z\left(i+1\right)=-x\left(i\right)-0.8y\left(i\right)+x{\left(i\right)}^{2}-x\left(i\right)z\left(i\right)+1 \end{array}$$

Assuming that there is a fixed point $\left(x^{*},y^{*},z^{*}\right)$ in Equation (1), the Jacobian matrix at this fixed point is shown in Equation (2):

(2) $$J=\left[\begin{array}{ccc} 0 & 1 & 0\\ 0 & 0 & 1\\ -1+2x^{*}-z^{*} & -0.8 & -x^{*} \end{array}\right]$$

The characteristic equation of the above equation is 3, as shown below:

(3) $$det\left(\lambda I-J\right)=\lambda^{3}+x^{*}\lambda^{2}-0.8\lambda +\left(1-2x^{*}+z^{*}\right)$$

According to the definition of fixed point, the unique fixed point $\left(x_{*}=y_{*}=z_{*}=0.3571\right)$ of the chaotic system can be obtained, and the eigenvalue of the system can be obtained according to Equations (2)and (3), as shown in Equation (4):

(4) $$\lambda =\{\begin{array}{c} \lambda_{1}=0.6495\\ \lambda_{2}=0.9949\\ \lambda_{3}=0.9949 \end{array}$$

**Figure 1:**
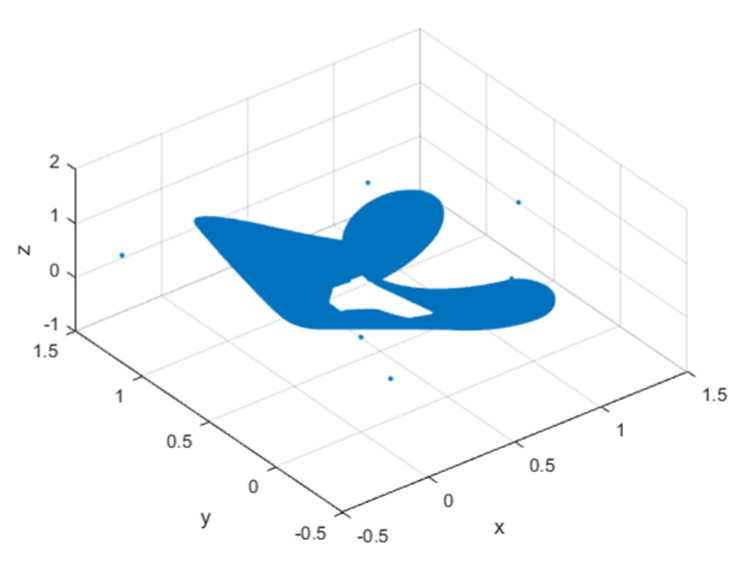
Chaotic attractor.

**Figure 2:**
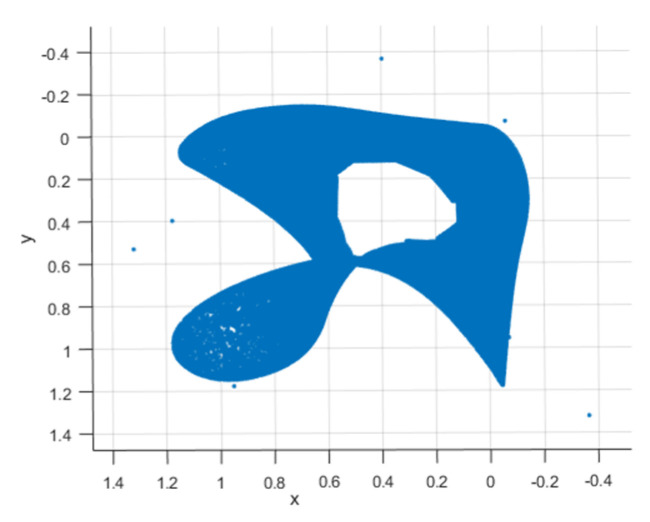
X-Y plan of chaotic systems.

It can be seen that the three eigenvalues at this fixed point are all within the unit circle, which proves that the system is a chaotic system with a hidden chaotic attractor with a stable fixed point. The attractor of its chaotic map is shown in Figure 1:

In the algorithm design, the x component of the chaos is selected for calculation. The projected image of the X-Y plane is shown in Figure 2:

### 2.2. Theory and Mathematical Model of Compressed Perception

There are two important principles in compressed sensing theory: sparsity and incoherence. Sparsity means that the signal contains a small number of non-zero values, other elements are 0, and the signal can be completely represented by these non-zero values. A two-dimensional signal X is assumed to have a size of N×N. The signal contains K non-zero values, where K<<N, which means that the signal can be sparsely represented under a sparse basis. The formula is shown as 5:

(5) $$X=\sum_{n=1}^{N}\psi_{n}s_{n}$$

where ψ is a sparse basis and S is the projection of X. After the original signal is sparse, the compressed sensing process can be represented by Equation (6):

(6) Y=φX=φψs

where φ is the measurement matrix a Y is called the measured value. The reconstruction process of compressed sensing is to obtain the original signal by solving the underdetermined equation on the basis of known measurement quantity and measurement matrix. The underdetermined equation should contain infinite solutions, but because the original signal is sparse, the only optimal solution can be obtained by solving the optimization problem. The equation of finding the optimal solution by using $L_{0}$ norm is shown as 7:

(7) $$\hat{\theta }=min{∥s∥}_{0}\;\;s.t.\;Y=\varphi \psi s$$

It can also be solved by using $L_{1}$ norm. The expression is shown as 8:

(8) $$\hat{\theta }=min{∥s∥}_{1}\;\;s.t.\;Y=\varphi \psi s$$

where $min{∥s∥}_{0}$ is the norm of $L_{0}$ and $min{∥s∥}_{1}$ is the norm of $L_{1}$. After obtaining the signal s, the original signal X can be obtained through an inverse transformation of the sparse process.

## 3. Encryption and Decryption Algorithms for Parallel Mixed Images Based on Compressed Sensing

This algorithm mainly focuses on the problem of mixed image, which is often encountered in image processing. An encryption algorithm is designed to encrypt the mixed image and verify whether the mixed image is encrypted effectively. This design adopts the way of parallel transmission to realize the efficient calculation of complex tasks. The low complexity distributed signal processing algorithm and encryption technology are fused together, which can effectively improve the efficiency of computing. At the reconstruction end, we improve the OMP algorithm and integrate the independent component analysis algorithm into the original reconstruction algorithm to realize the restoration and separation of mixed images. At the same time, we use the initial value sensitivity and pseudo-randomness of chaotic systems in the algorithm, which can greatly improve the key space.

### 3.1. Logic Circuit and Parallel Algorithm Design

In this paper, we design a simple structure of the digital logic circuit. We can, through the circuit, generate a string of length n binary signal in the process of the generation of chaotic matrix. There is a need to use a string of binary signal to change every initial value of the matrix because chaotic systems have the features of initial value sensitivity—after initial value change generated each chaotic matrix is not the same. In past studies, some scholars proposed a CS strategy based on half tensor product (STP), which can greatly reduce the storage space occupied by a measurement matrix and maintain high reconstruction quality [32]. In the algorithm proposed in this paper, the measurement matrix does not need to be transmitted to the receiver as a key. The receiver only needs to use the structure of the logic circuit and the initial value of the chaotic matrix to reproduce the measurement matrix. It can effectively reduce the storage space of measurement matrix, improve the memory utilization rate and reduce the amount of data.

**Figure 3:**
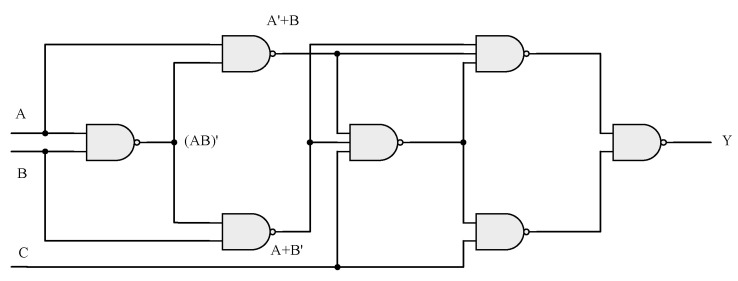
Signal generation logic circuit.

In order to make the design of logic circuit can be more concise, at the same time also can meet the demand of the function of the algorithm, we adopt a three variable parity detection circuit, the circuit is composed of gate in the structure, the advantage of this circuit is simple, portable, convenient as part of the process of encryption and decryption keys. For example, if this paper needs to generate a set of signals such as 10010110, the design method shown in Figure 3 can be adopted: 

The above circuit is a parity detection circuit with three variables. When there are even numbers of 1s and all 0s in the input variable, the output is 1; otherwise, the output is 0. Therefore, the order of output sequence can be determined by adjusting the value of the three input terminals. The mathematical expression of this circuit is shown in Equation (9):

(9) Y=A′B′C′+A′BC+AB′C+ABC′

According to the above principle, we use the variable data in Table 1 to generate sequence signals:

Where A, B, and C are the initial input terminals, binary signals of arbitrary length can be obtained by setting different initial input values, and signals of corresponding length can be generated according to actual needs. In this paper, eight-bit binary sequence is generated to adjust the initial parameters of the chaotic system. Compared with the logic circuit designed before, this circuit has the advantage of simple structure, easy to reproduce the key at the receiving end, and no longer limited to the digit limit of the circuit device. We can generate acyclic binary sequence of arbitrary length as required.

**Figure 4:**
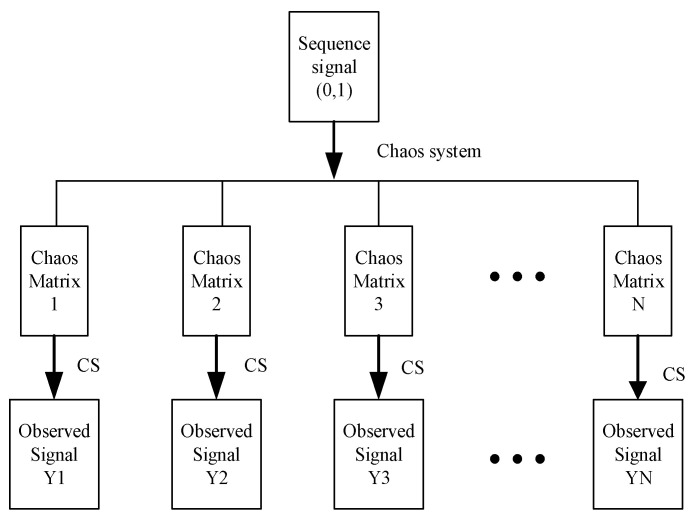
Parallel encryption process.

**Figure 5:**
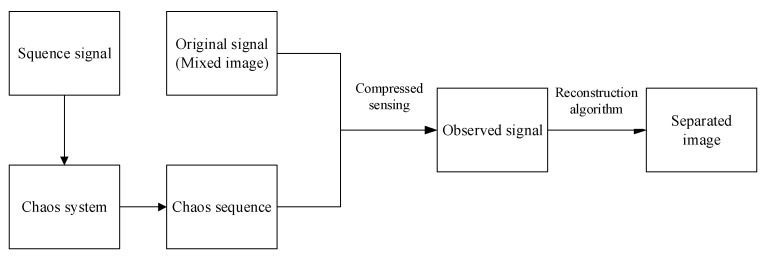
Schematic diagram of the algorithm.

After generating binary sequences, this algorithm uses the sequences to control the initial parameters of chaotic systems and generates different chaotic systems as the measurement matrix of compressed sensing process. In this paper, the sparse mixed signal is evenly cut into eight pieces, each of which has a size of 256×32. The eight pieces are compressed and encrypted at the same time, which can effectively improve the encryption transmission efficiency and computational complexity. The initial parameters are controlled by binary signals to generate chaos matrix, which is used as measurement matrix for parallel compression and encryption, as shown in Figure 4:

As shown in the Figure 5, binary signals are used as key parameters to control the generation process of chaotic matrix. To encapsulate it into after generating chaotic signal measurement matrix, and the image compression of sparse after sampling, due to the block sample will make the energy is concentrated in the process of preservation, in order to overcome this problem, we have compressed sampling signal to diffusion and scrambling after operation, the energy is uniformly distributed in the image, the observation signal Y.

### 3.2. Principle of Encryption Algorithm

The algorithm designed in this paper is divided into two parts: encryption and decryption. The signal generator and parallel transmission principle introduced in 3.1 are used in the design process. The generated binary signal and the initial parameters of the chaotic system can be transmitted as the key, which can reduce the computational resources consumed in the key transmission. The schematic diagram of the overall algorithm is shown in Figure 5:

The chaotic map used in this algorithm is the 3D chaotic map introduced in the second part of this paper. The generated chaotic signals are encapsulated as measurement matrix. In order to give consideration to the computing efficiency and the consumption of computing resources, we divide the image of 256*256 into eight pieces, encrypt and transmit them, respectively, and then combine them into one picture at the receiving end. The image part of the original signal is a mixed image, and the mixing mode of the image is unknown. The specific implementation processes of encryption algorithm are as follows:

1. Select two images with a dimension of and mix them in two different ways. The mixing method is unknown, and it does not participate in the subsequent decryption process as a key.
2. A series of binary sequences are generated by the designed signal generator, and the initial value sensitivity of the chaotic system is used to fine-tune the initial value of the selected chaotic system to generate different pseudo-random signals. The chaotic signals used in this paper are generated by a three-dimensional mapping chaotic system with fixed points.
3. The generated chaotic signal is encapsulated into a compression matrix, whose dimension is determined by the compression ratio.
4. Divided the mixed image into eight pieces of equal size and carried out the compressed sensing sampling process on them, respectively. This process utilized the idea of parallel computing and carried out transmission simultaneously. After sampling, the eight ciphertext images are synthesized into one image, which is scrambled and diffused to obtain the ciphertext image.

### 3.3. Decryption Algorithm Introduced

The decryption algorithm is roughly the inverse operation of the encryption algorithm. After the encryption is completed, the image is transmitted to the receiving end, which needs to recover the measurement matrix used in the encryption process through the known key. The image is reconstructed by means of measurement matrix. The specific algorithm steps are as follows:

1. The reverse diffusion operation is carried out on the observed signal to obtain the ciphertext image after sampling by compressed sensing.
2. The traditional OMP reconstruction algorithm in the compressed sensing framework is used to restore the image, and the recovered image is a sparsely mixed image.
3. The original mixed image is obtained by using inverse wavelet transform.
4. Since the way of image mixing is unknown, effective image information cannot be separated by inverse operation. Therefore, the FAST ICA algorithm based on fixed-point recursive algorithm is introduced here. Through this Fast optimization iterative algorithm, we can estimate the mixed signals to achieve the separation of mixed images. FastICA is also known as the fixed-point algorithm. It’s based on fixed point recursion. It works for any type of data. This algorithm belongs to a kind of neural network algorithm, but the difference with the general algorithm is that the algorithm can carry out batch processing.

FastICA algorithm is based on fourth order cumulant, based on maximum likelihood, maximum negative entropy and other forms. Compared with ordinary ICA algorithm, this algorithm has faster convergence speed. Moreover, FastICA algorithm is easier to use because it does not need to select step size parameters. Here’s how FastICA works:

In the process of separation, we first remove the mean value of the observed signal and subtract the mean vector in the signal to make the observed signal zero mean variable. Due to the correlation of the data, it is necessary to whiten it to simplify the extraction process of subsequent independent components. The whitening process is shown as follows:

(10) $$z\left(t\right)=W_{0}x\left(t\right)$$

where $W_{0}$ is the albino transformation matrix, Z is the albino vector. The whitening operation can reduce the parameters to be estimated. In the process of estimating multiple independent components, it is necessary to extend the maximum non-Gaussiality method and ensure that different signals are separated by compression orthogonalization. We can measure non-Gaussiality in terms of entropy, usually in the modified form of entropy, negative entropy. In the separation process, the non-Gaussiality measure of the separation results can be used to express the mutual independence between the separation results. When the non-Gaussiality measure reaches the maximum, it indicates that the separation process of each independent component has been completed. The definition of negative entropy is as follows:

(11) $$N_{g}\left(Y\right)=H\left(Y_{Gauss}\right)-H\left(Y\right)$$

where $\;Y_{Gauss}$ is a Gaussian random variable with the same variance as Y, and H(.) is the differential entropy of the random variable.

(12) $$H\left(Y\right)=-\int^{\;}p_{\gamma }\left(\xi \right)\mathrm{lg}p_{\gamma }\left(\xi \right)d\xi$$

According to information theory, among random variables with the same variance, Gaussian random variables have the largest differential entropy. The fast ICA learning plan is to find a direction so that $W^{T}X\left(Y=W^{T}X\right)$ has maximum non-Gaussian properties. The maximum approximation of negative entropy of $W^{T}X$ can be obtained by optimizing $E\left\{G\left(W^{T}X\right)\right\}$. According to Kuhn-Tucker condition, under the constraint of $E\left\{{\left(W^{T}X\right)}^{2}\right\}={∥W∥}^{2}=1$, the optimal value of $E\left\{G\left(W^{T}X\right)\right\}$ can be obtained at the point satisfying the following equation.

(13) $$E\left\{Xg\left(W^{T}X\right)\right\}+\beta W=0$$

Here β is a constant value $\beta =E\left\{{W_{0}}^{T}Xg\left(W_{0}^{T}X\right)\right\}$, $W_{0}$ is an optimized value of W. The left-hand function of the above equation is expressed by F, and its Jacobian matrix can be obtained as follows:

(14) $$JF\left(W\right)=E\left\{XX^{T}g\prime \left(W^{T}X\right)\right\}-\beta I$$

The iterative formula of the simplified Fast ICA algorithm is shown in Equation (10):

(15) $$\begin{array}{c} W^{*}=E\left\{Xg\left(W^{T}X\right)\right\}-E\left\{g\prime \left(W^{T}X\right)\right\}\;W\\ W=\frac{W^{*}}{∥W^{*}∥} \end{array}$$

where  E[.] is the mean operation, g(.) is the nonlinear function, W is the estimated value, and $W^{*}$ is the new value, which has higher stability than W.

### 3.4. Algorithm Simulation Verification

**Figure 6:**
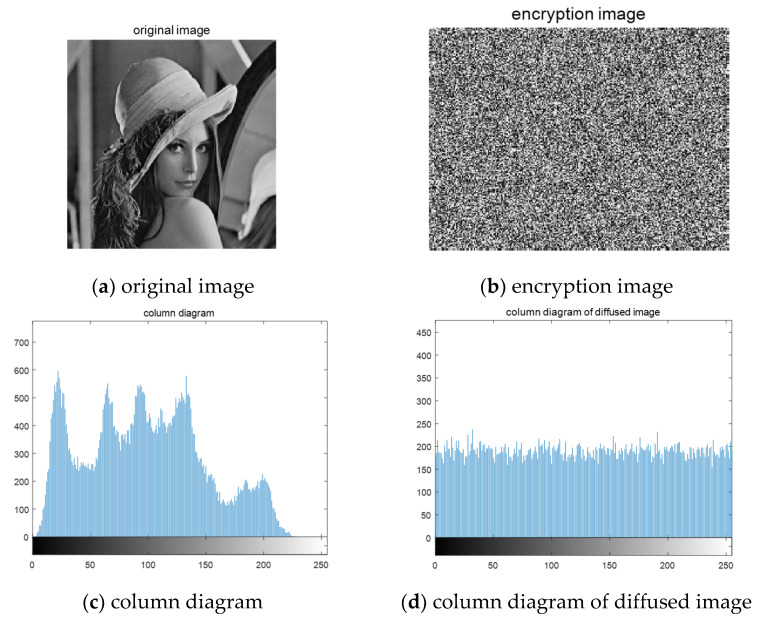
Encrypted image and its histogram.

**Figure 7:**
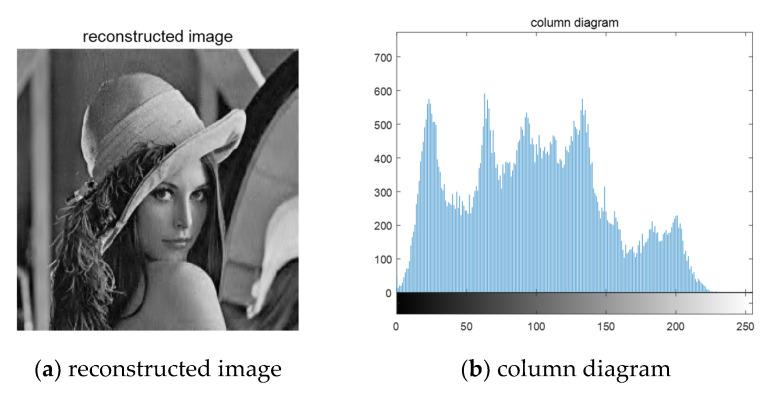
Restored image and its histogram.

In order to verify the effectiveness of the above-proposed algorithm, we first encrypt and reconstruct the unmixed normal images. We select a set of  256×256 grayscale image "Lena" from the standard image library and use DWT wavelet transform to sparse the images. The initial value of 3d chaotic system is x(1)=0.12, y(1)=−0.06, z(1)=−0.07, When the binary signal is 1, the chaotic initial value is superimposed with $10^{-6}$, and when the binary signal is 0, the initial value is superimposed with $\;10^{-8}$. A total of eight different chaotic matrices need to be generated. After generating the chaotic signal, it is encapsulated into a measurement matrix, and eight original images are encrypted in parallel using the compressed sensing algorithm. The encryption results are shown in Figure 6:

Through the above image, you can see, when we use the algorithm to the normal image encryption, encryption image is a snowflake, which does not contain any useful information related to the original information, the original image histogram pixel distribution area is visible, and cipher-text uniform distribution histogram of the image pixels, each pixel area, cannot see the attacker can’t get any useful information, suggesting that encrypted with normal images of the proposed algorithm can be used effectively. The decryption process can be regarded as the reverse operation of the encryption process. First, the cipher-text image is anti-diffused, the chaotic system is generated by the key, the measurement matrix is obtained, and then the original signal is reconstructed by solving the optimization problem. Figure 7 shows the decrypted image and its histogram.

It can be seen from Figure 7 that this algorithm can reconstruct the unmixed image, and the reconstructed image can clearly reflect the useful information of the original signal. The histogram of Figure 7b is basically similar to the pixel distribution of Figure 6c, so we can conclude that this algorithm is effective for ordinary images.

## 4. Analysis of Encryption and Decryption Algorithm and Separation of Mixed Images

The main purpose of this paper is to process mixed images. In reality, there are many situations to process mixed images, such as image overlay in image software, lens transformation and other pollutions resulting in image mixing. This paper intends to sample and encrypt the mixed image in an unknown way to verify whether the algorithm designed in this paper is suitable for the mixed image and whether the image can be effectively separated after image reconstruction at the receiving end.

### 4.1. Analysis of Encryption and Decryption Algorithm and Separation of Mixed Images

**Figure 8:**
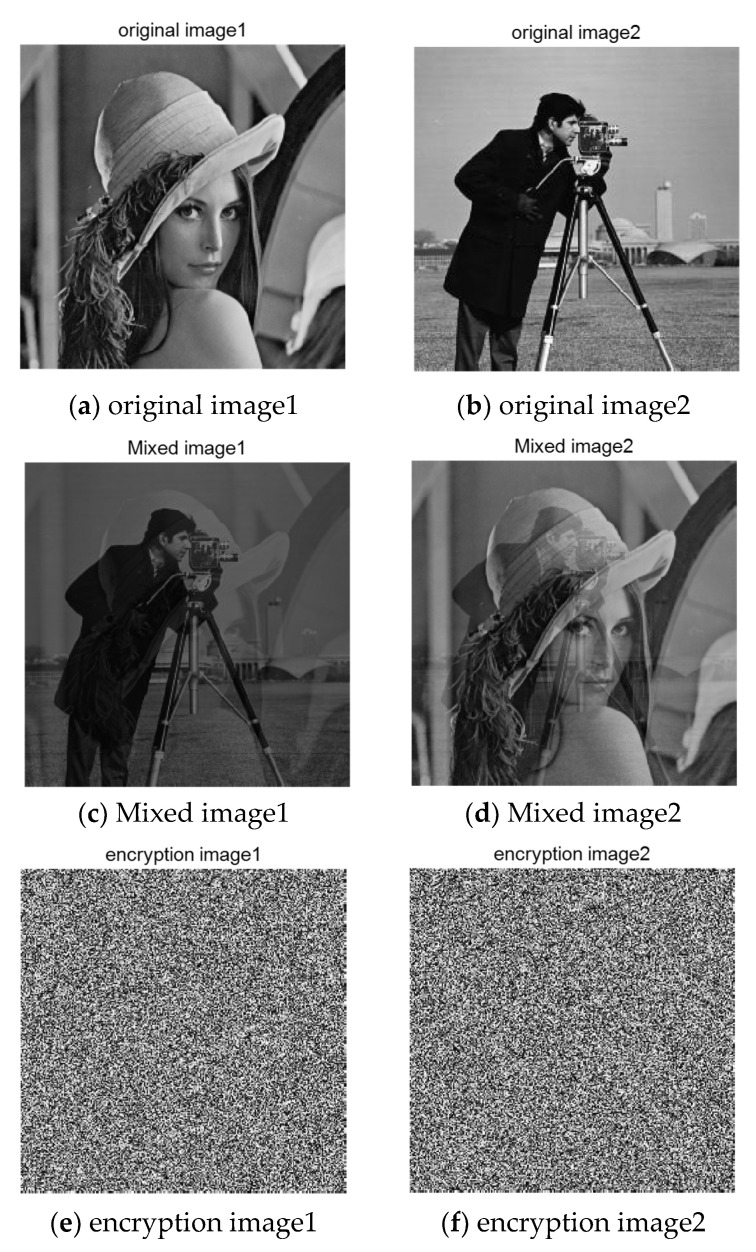
Mixed image encryption.

**Figure 9:**
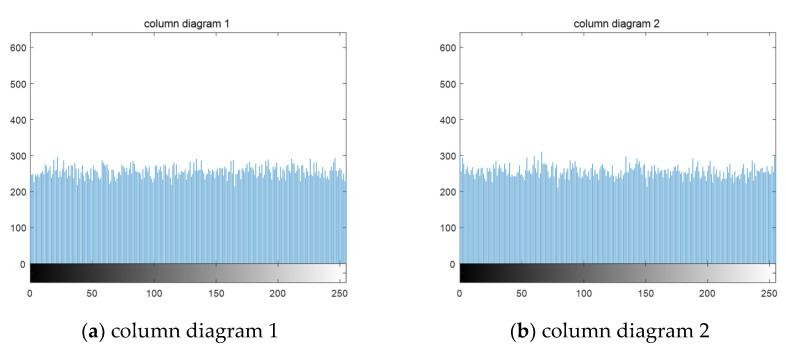
Cipher-text image histogram.

The mixed image used in this section is obtained by mixing 256×256 grayscale image “Lena” and “Cameraman”. Since Fast ICA algorithm is needed in the separation process, encryption, and reconstruction of the two arbitrarily mixed images are needed. The sparse image is divided into eight equal parts by wavelet transform, and each subblock is compressed and sampled by chaos matrix. First, judge whether the algorithm in this paper can effectively encrypt the mixed image. The simulation results are shown in Figure 8:

Figure 8a,b, respectively, represent the original images before mixing. After linear mixing in an unknown way, the two mixed images are obtained, such as Figure 8c,d. In the experiment of this paper, the two mixed images are the original signals, and the images before mixing are used to verify the image effect after separation. It can be seen that after the compression and encryption of the mixed image by the algorithm in this paper, the cipher-text is snowflake-like and any valid information related to the plain text cannot be observed. The following is the histogram of cipher-text image drawn, as shown in Figure 9:

According to the distribution of the histogram, we can see that the pixels of the cipher-text image are evenly distributed, indicating that we successfully hide useful information. In the case of unknown key, the thief cannot get any information related to the plain text from the cipher-text image. In the case that useful information cannot be differentiated from a subjective perspective, this section analyzes objective quantitative indicators such as information entropy, which is a concept used to measure information in information theory. The more orderly a system is, the lower the information entropy will be; conversely, the higher the information entropy is, the greater the uncertainty of variables will be, and the greater the amount of information needed to understand the specific content will be. Table 2 calculates the information entropy of two cipher-text images under different compression ratios:

It can be seen that the entropy of the two cipher-text images listed in the above table is still close to 8 despite the impact of compression rate. From the perspective of entropy, the encryption part of this algorithm is effective. Literature [24] proposed a fast and efficient multiple-image encryption algorithm. The algorithm uses a chaotic system to generate sequences to replace subblocks in the image. The image is encrypted using Arnold Cat Map and several operations, including XOR and cyclic shift. Wen proposed a CS strategy based on a semitensor product (STP) [32]. The comparison results with other literature are as follows in Table 3:

By integrating visually secure image encryption techniques into the system, the final encrypted image can be visually aligned with the normal image. As can be seen from the comparison in Table 3. The entropy value of the proposed algorithm is close to 8, and both of them can realize effective encryption.

**Figure 10:**
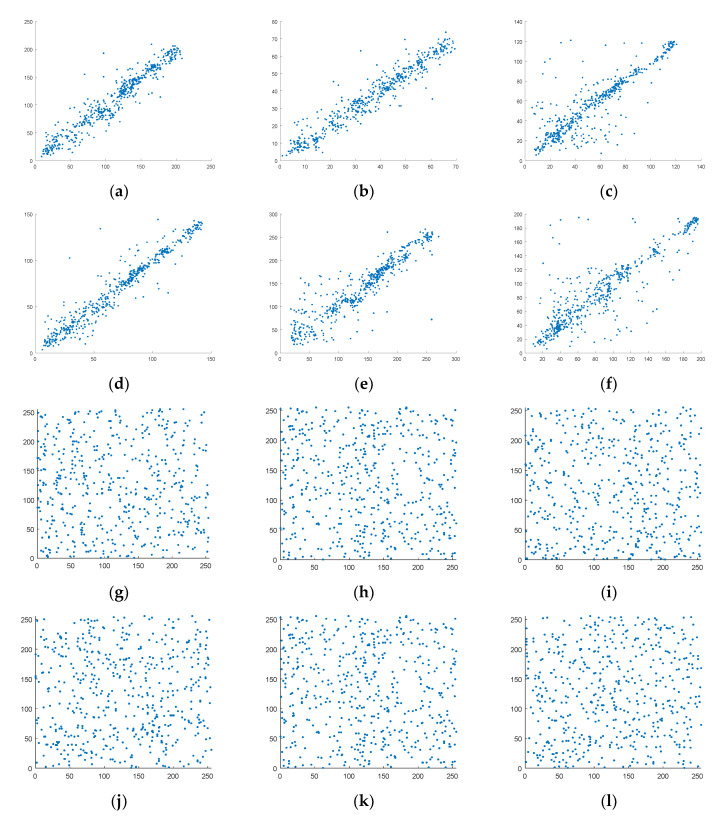
Correlation distribution of adjacent pixels (**a**–**c**) are the correlation distribution of the first mixed image, (**d**–**f**) is the correlation distribution of the second mixed image, (**g**–**i**) is the correlation distribution of the first cipher-text image, (**j**–**l**) is the correlation distribution of the second cipher-text image.

The correlation of adjacent pixels describes the correlation between pixel values at adjacent locations in an image. A qualified encryption algorithm should try to reduce the correlation between adjacent pixels. We analyzed from the horizontal, vertical, and diagonal directions. When the compression rate is approximately equal to 0.7, the distribution of pixel points is first given, as shown in Figure 10:

It can be seen that, despite the mixing, the correlation between pixels in the original signal is still high, while the pixel points of cipher-text image are evenly distributed within the pixel interval, and the correlation is very weak. On the basis of Figure 10, we calculated the correlation coefficient of cipher-text images as shown in Table 4:

### 4.2. Image Reconstruction Results and Separation Performance

**Figure 11:**
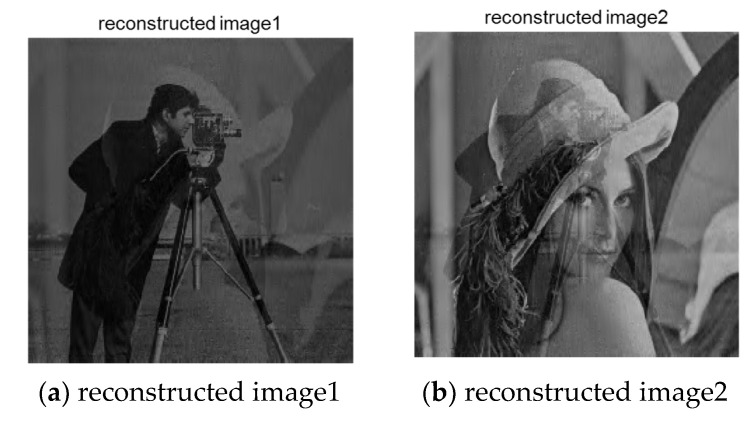
Reconstructed image.

The signal recovery process is divided into two stages. To achieve the final image separation, the reconstruction process of mixed signals should be completed first. The image recovered by the reconstruction algorithm mentioned above is shown in Figure 11. It can be seen that, although the image is still in the mixed state, the algorithm successfully separates the encrypted mixed image.

**Figure 12:**
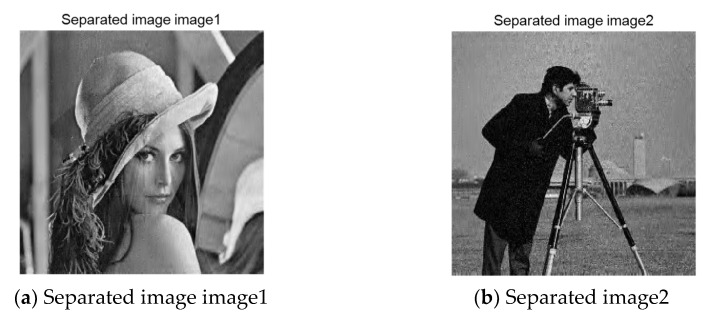

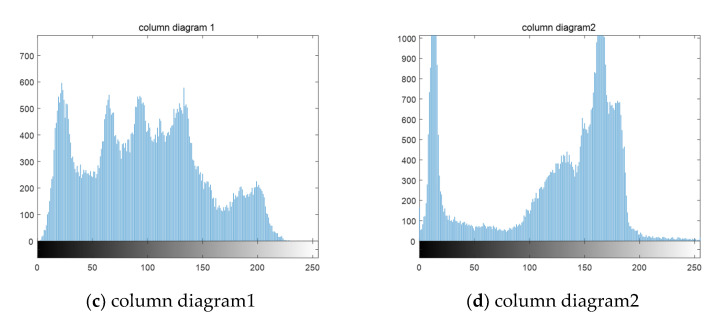
Separate images and their histograms.

After the mixed image is obtained through the reconstruction algorithm, the two images processed by Fast ICA algorithm described above are used to verify whether the algorithm in this paper can successfully achieve the encryption and recovery of the mixed image. The result after the separation is shown in Figure 12. As can be seen from the figure, the two pictures are effectively separated. Subjectively, we can judge the information to be expressed in each picture.

According to the above image and histogram, after the algorithm designed in this paper, the two images “Lena” and “Cameraman” are separated, respectively. The original information of the separated image is clearly visible, the histogram distribution is similar to the original image, and the pixel distribution range is relatively clear. We believe that the hybrid image can be decrypted and separated effectively after the algorithm reconstruction. The PSNR can describe the distortion of the signal, and the higher the PSNR of the image, the lower the distortion degree will be. Table 5 lists the PSNR of the two separated images under the condition of different compression rates.

Structural similarity is an important index to measure the similarity degree of two images. When there is a strong correlation between the images, the value of structural similarity is larger. When the correlation between images is weak, the value of structural similarity is small. The value range of structural similarity is 0 to 1. When the similarity is close to 1, the two pictures are more similar; otherwise, the difference between the two pictures is greater. Table 6 shows the degree of similarity between the two images and the original image after the reconstruction separation algorithm:

As can be seen from the table, although the mixing of images has a certain impact on the experimental results, the structural similarity between the separated image and the original image is greater than 0.6 when the compression ratio is greater than 0.5, indicating that this algorithm can effectively reconstruct and restore the encrypted image of the mixed image. In practical application, the appropriate compression ratio can be selected according to the needs.

**Figure 13:**
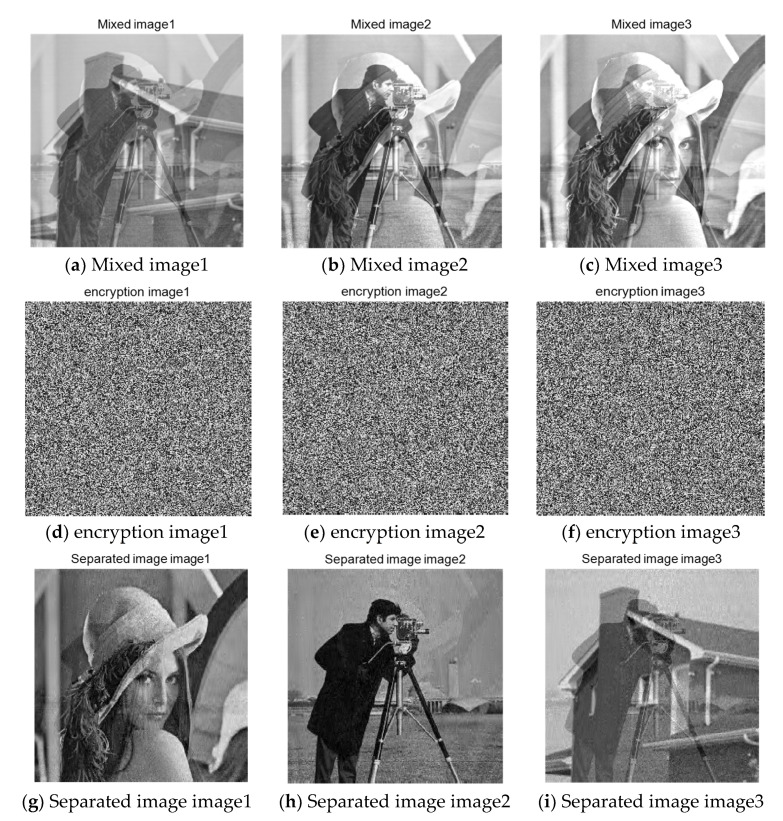
Encryption and decryption results of three mixed images.

As can be seen from the table, the peak value signal-to-noise ratio increases with the increase of compression ratio, due to the resulting image after image encryption, refactoring and separation of a series of steps, the peak value signal-to-noise ratio have been affected, but we can see the image of the separated when compression ratio is close to 0.7, PSNR is close to 30, image has good separation effect. Simulation analysis was carried out on the mixed image of the two images above, and the image made from the mixed image of the three images was verified below to determine whether it could be encrypted and reconstructed using the algorithm proposed in this paper. In the original two mixed images, the “house” image in the standard gallery is mixed. The size of the image is 256×256. The mixed image, encrypted image, and separated image are respectively shown in Figure 13:

It can be seen from the above figure that the mixed image containing three images can be effectively recovered, and the encryption effect is snowflake, which verifies that the algorithm in this paper can successfully realize the encryption sampling, transmission, and reconstruction separation of the mixed image. In practical application, the appropriate compression rate can be selected to encrypt according to the needs, so as to simplify the computational complexity and speed up the computational efficiency.

### 4.3. Safety Analysis

**Figure 14:**
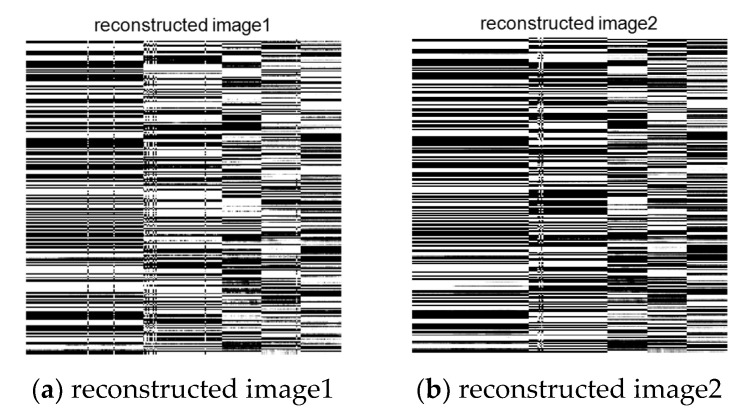
Key sensitivity analysis.

Due to the sensitivity of the encryption algorithm to the key, small changes in the key will directly lead to the failure of the decryption process. Figure 14 shows the decryption result of the image mixed by two images when the chaotic initial value changes by $10^{-16}$. As can be seen from the figure, when the initial value of chaos changes extremely slightly, it still leads to the failure of decryption results, which indicates that this algorithm has a high key sensitivity, and the advantage of this performance is that it can resist violent attacks.

Effective image encryption scheme should be large enough key space to combat violent attacks, the algorithm proposed in this paper, considered in only nine chaotic system control parameters, the influence of the IEEE 754 according to international standards, in order to simplify the comparison, will represent positive indexes section, including double-precision floating-point type of valid number is 52. After calculation, the key space in this paper is at least $2^{52\times 9}=2^{468}$, which means that the attacker needs a second attack to build the correct matrix. Table 7 lists the comparison between the key space of this algorithm and other literature. It can be seen that the key space of this algorithm is higher than that of similar algorithms, and it is almost impossible for illegal attackers to find the correct key of the algorithm through violent attacks, so the encryption algorithm can resist violent attacks.

The Number of Pixels Change Rate (NPCR) or the normalized Average Changing Intensity (UACI) can be used to measure whether the encryption algorithm is able to resist differential attacks. For the 8-bit grayscale image, the expected values of NPCR and UACI are: NPCR = 99.6094% and UACI = 33.4635%. The experimental results are shown in Table 8:

As can be seen from the table, the NPCR and UACI calculated by the image encrypted and separated by the algorithm in this paper are close to the ideal values, slightly higher than the values in the references, so the algorithm in this paper has a better ability to resist differential attacks.

**Figure 15:**
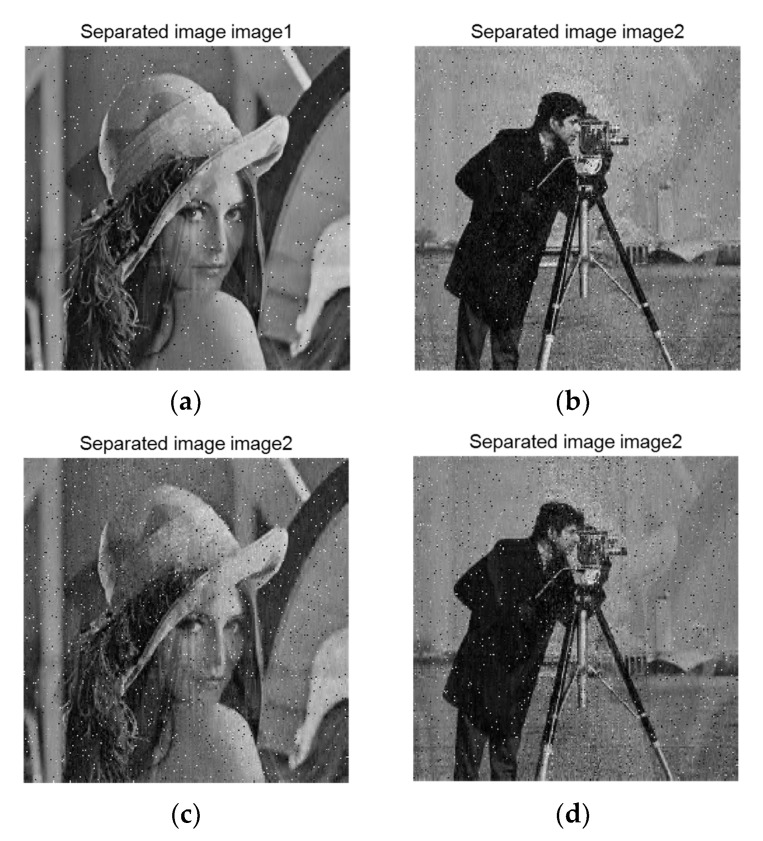
Robust to noise attack. (**a**) decrypted image ‘lena’ from the cipher image distorted by Salt and pepper noise with intensity of 0.01. (**b**)decrypted image ‘cameraman’ from the cipher image distorted by Salt and pepper noise with intensity of 0.01.(**c**) decrypted image ‘lena’ from the cipher image distorted by Salt and pepper noise with intensity of 0.02. (**d**) decrypted image ‘cameraman’ from t

In the process of transmission, the encrypted image will be affected by noise attack or noise interference, which will make it change, affect the effect of the restored image and even lead to the failure of decryption. Therefore, we need to evaluate the robustness of the proposed algorithm against noise. In order to evaluate this feature, we added noise to the encrypted image. In this experiment, we added salt and pepper noise with intensity of 0.01 and 0.02, respectively. The results after reconstruction and separation are shown in the Figure 15.

As can be seen from the figure, under the attack of noise with different intensity, the quality of decryption decreases with the increase of noise intensity, and some noise remains in the image. However, even under the interference of noise, the original signal can still be separated effectively, and the main information of the image can be identified. Therefore, we can conclude that the algorithm has a certain robustness to noise.

The algorithm in this paper takes about 0.5s to encrypt a single mixed image. Table 9 lists the time comparison between the proposed encryption algorithm and similar literature. In the literature [24], a fast and efficient multi-image encryption algorithm based on chaotic systems is proposed. In the literature [39], a multi-image encryption algorithm based on bit-plane decomposition and chaotic mapping is proposed. Combined with the decryption and separation process, the overall running time of the algorithm is about 14s. Compared with the general encryption algorithm, the processing mode of parallel transmission designed in this paper can effectively improve the computational efficiency. By calculating the basic statements in the algorithm, the time complexity of the proposed algorithm is O(n).

## 5. Conclusions

In this paper, a parallel encryption scheme based on compressed sensing framework is designed to study the mixed images encountered in real image processing. From the perspective of information security and transmission efficiency, this scheme combines chaotic signals with compressed sensing theory to compress and sample the mixed images. At the same time, the design of parallel algorithm can realize efficient computation of complex tasks and encrypt and transmit the original information in blocks at the same time, which can reduce the computation time of data to the maximum extent. The decryption process integrates Fast ICA algorithm on the basis of the traditional OMP algorithm to realize the reconstruction and separation of mixed images. Through experimental analysis, it can be seen that the algorithm designed in this paper can encrypt mixed images with good encryption effect. At the same time, because the algorithm contains more control parameters, it can greatly improve the key space of the algorithm to resist violent attacks. After decryption and separation, the image content is clear, clear-cut, peak signal-to-noise ratio, and other parameters can meet the requirements of the decryption algorithm. Therefore, the parallel image encryption technology based on compressed sensing designed in this paper can encrypt and separate the mixed image and meet the security requirements of the encryption algorithm.

## Figures and Tables

**Table 1:** Truth value table of logical circuit.

A	B	C	Y
0	0	0	1
0	0	1	0
0	1	0	0
0	1	1	1
1	0	0	0
1	0	1	1
1	1	0	1
1	1	1	0

**Table 2:** Entropy of encrypted images.

Entropy	Compression Ratio						
	0.4	0.5	0.6	0.7	0.8	0.9	1
Cipher image1	7.9973	7.9975	7.9971	7.9973	7.9968	7.9975	7.9973
Cipher image2	7.9972	7.9971	7.9972	7.9973	7.9968	7.9979	7.9972

**Table 3:** Entropy comparison of different algorithms.

	Cipher image1	Cipher image2	Ref. [24]	Ref. [32]
Entropy	7.9973	7.9973	7.9995	7.4682

**Table 4:** Correlation coefficients of adjacent pixels.

Algorithm	Horizontal Direction	Vertical Direction	Diagonal Direction
Proposed algorithm (Cipher image1)	0.0271	0.01308	−0.00427
Proposed algorithm (Cipher image1)	−0.0398	0.0151	0.0042
Ref. [30]	−0.03851	0.002318	0.00853
Ref. [33]	0.27304	0.017541	0.0069054
Ref. [34]	0.029811	−0.035907	0.0052338

**Table 5:** Peak SIGNAL to noise ratio.

**PSNR**	**Compression Ratio**						
	**0.4**	**0.5**	**0.6**	**0.7**	**0.8**	**0.9**	**1**
Separation image “lena”	23.3846	27.7247	29.6284	30.2927	31.0976	31.8893	32.0102
Separation image “cameraman”	24.0628	27.7791	30.0369	30.7298	31.8524	32.3356	32.5290

**Table 6:** Structural similarity between original image and separated image.

**SSIM**	**Compression Ratio**							
	**0.3**	**0.4**	**0.5**	**0.6**	**0.7**	**0.8**	**0.9**	**1**
Separated image “lena”	0.2237	0.4105	0.6641	0.7933	0.8312	0.8864	0.9013	0.9007
Separated image “cameraman”	0.1904	0.3392	0.6247	0.7419	0.8023	0.8578	0.8996	0.9001

**Table 7:** Key space comparison.

Algorithm	Proposed Algorithm	Ref. [35]	Ref. [32]	Ref. [36]	Ref. [37]
Key space	2^468^	2^460^	2^32^	2^356^	2^194^

**Table 8:** NPCR and UACI analysis of the isolated images.

Index	Our Scheme	Ref. [38]	Ref. [36]
NPCR(%) “lena”	99.6143	99.6075	99.6024
NPCR(%) “cameraman”	99.6205		
UACI(%) “lena”	33.4641	33.4195	33.4331
UACI(%) “cameraman”	33.5016		

**Table 9:** Encryption time and Computational Complexity.

Algorithm	Algorithm in this Paper	Ref. [24]	Ref. [39]
Encryption time(s)	0.5	0.95	9.656
Computational complexity	O(n)	---	---
